# Synchronized Hardware-Registered VIS-NIR Imaging Spectroscopy and 3D Sensing on a Fresco by Botticelli

**DOI:** 10.3390/s21041287

**Published:** 2021-02-11

**Authors:** Jana Striova, Luca Pezzati, Enrico Pampaloni, Raffaella Fontana

**Affiliations:** National Institute of Optics of the National Research Council of Italy, INO-CNR, Largo Enrico Fermi 6, 50125 Firenze, Italy; jana.striova@ino.cnr.it (J.S.); enrico.pampaloni@ino.cnr.it (E.P.); raffaella.fontana@ino.cnr.it (R.F.)

**Keywords:** heritage science, 3D acquisition, reflectance imaging spectroscopy, multi-spectral reflectography, mural painting, registered data

## Abstract

We discuss a synchronised sensing technique for the analysis of painted surfaces of frescos. Specifically, the performance of Visible-Near Infrared (VIS-NIR) Reflectance Imaging Spectroscopy (RIS) synchronized with three-dimensional (3D) acquisition is demonstrated in the study of a detached mural painting by Alessandro Botticelli. Synchronized sensing generates georeferenced data for simplified data treatment and interpretation. We show how such output data can provide key information to interpret important fresco surface and subsurface features (e.g., painting technique, material composition, pentimenti).

## 1. Introduction

Optical analytical tools provide crucial information about paintings and artworks’ materials and techniques. Innovative non-contact (and non-invasive) optical methods are under constant development, with controlled levels of radiation emission allowing the safekeeping of examined objects. Multisensorial imaging methods play an ever-increasing role in the examination of painted surfaces, capable to deliver complementary information on the entire artwork’s surface and stratigraphy. The visible-near infrared (VIS-NIR) imaging scanning device, coupled with a 3D acquisition sensor, described here, exploits the property of IR radiation to penetrate the pictorial layers. The spectral information carried by visible (VIS) and infrared (IR) radiation reveals properties and composition of the probed matter, and the laser distance sensor retrieves the surface topography. While the first instruments for IR reflectography collected the radiation in a single, broad spectral range [1], the method gradually evolved, starting from the mid 1990’s, into the multi/hyper spectral (MS/HS) modality, derived from Earth remote sensing techniques [2]. The great potential of MS/HS reflectance imaging spectroscopy (RIS) in the analysis of artworks has been recognized by the heritage science community, today including a large number of research groups active in this field [3,4,5,6,7,8,9,10,11,12,13,14,15,16,17,18]. A detailed account of the evolution and progress, as well as of the fundamental physical principles of RIS and systems involved, is provided in a recent review [19]. Key advantage of RIS is the integration of the spectroscopic and the imaging approaches for disclosing hidden features, for differentiation/classification of various components and their spatial distribution in a non-invasive way.

The point-scanning approach, based on the detection of radiation reflected from painted surfaces by boustrophedon movement of a single photodiode, was introduced in the 1990’s by a group of researchers at the National Institute of Optics (INO, now in the Italian National Research Council, INO-CNR) [8,18,20]. Such approach permits a very easy alignment and registration of geometrically-corrected images, at the cost of longer acquisition times. The technique evolved across several prototypes, leading to the current multi-spectral system producing 16 bands in the 380–780 nm VIS and 16 bands in the 780–2500 nm NIR spectral ranges [18]. Such an extended spectral coverage offers several advantages, given an increasing transparency of most of pictorial materials with increasing wavelength (multi-spectral infrared reflectography), ending up in a better visibility of the preparatory drawing (the underdrawing) usually found under the colour layers of ancient paintings. The capacity to measure in the visible, the near-infrared and the shortwave regions makes RIS a powerful technique for characterizing artwork materials. Advanced methods of data analysis facilitate data classification, pattern recognition and prediction derived from existing data. The RIS method alone has a robust but non-exhaustive capacity for the characterization of the composite painted systems. Therefore, RIS can be coupled with other spectroscopic or non-spectroscopic sensors to measure a larger variety of properties [19]. The association of multimodal sensors producing hardware-registered (multimodal) datasets avoids the necessity to employ a subsequent software data fusion methodology. The benefits of the synchronized registered 3D scanning and MS imaging are mainly in eliminating the necessity of two separate instruments, reducing the acquisition time and computational capacity needed for processing and co-registering 3D data on MS images. To the best of our knowledge, the only system recording simultaneously 3D data with multispectral imaging in the field of heritage science is the PRISMS (Portable Remote Imaging System for Multispectral Scanning) devised by the Nottingham Trent University, designed for the remote sensing of large painted surface such as wall paintings. As compared to our scanner, PRISMS operates in a completely different optical set-up and acquires in a more limited (i.e., 400–900 nm) spectral range [21].

The extraction of three-dimensional (3D) information is of interest in many application fields, including monitoring of the artwork cleaning operations and evaluation of its conservation state and history [22,23,24]. Differences in principles and characteristics of 3D techniques involve various factors such as cost, acquisition accuracy, speed and range. Indeed, diverse systems, sensors, and techniques have been developed in terms of their physical size, robustness, weight, interfacing option, reliability, process requirement, high-resolution, price, and power consumption to meet the needs of a specific application field. Laser triangulation, involved in the discussed device, can be implemented using off-the-shelf components, representing a relatively low cost and fast approach [25].

By showcasing significant results obtained on the detached mural painting *Sant’Agostino nello Studio*, by the Florentine Renaissance Master Sandro Botticelli, this article provides insights into how the combined RIS and 3D sensing enable the disclosure of hidden features in the artwork, of the chemical mapping of the constituent materials and is capable to reveal details of the artwork preparation and realization process.

## 2. Materials and Methods

### 2.1. VIS-NIR Multispectral Scanner

The expertise gained in the last 30 years with the scanning device for short-wave infrared (SWIR) imaging in a single broad band led to the actual version of multispectral scanner, having 32 bands in the spectral range from 380 to 2500 nanometres. Specifically, an XY(Z) scanning system moves both the lighting system and the collecting optics, which are set to a 45°/0° illumination/detection geometry, following CIE indications for non-contact reflectance colour measurements [26]. The scanning system consists of two orthogonally mounted XY stages having a maximum run of 1000 mm and of a Z stage with a total run of 100 mm to keep the optical head in focus while scanning the surface.

The lighting system is composed of two low-voltage current-stabilized halogen lamps, equipped with aluminium back-reflectors whose beam divergence is ±5° and of two narrow-spot white LEDs, with beam divergence of about ±4.5°. The uniformly irradiated area on the painting surface is about 5 cm^2^. The irradiance as measured on the painting surface in standard illumination conditions is about 105 kLux (from the LEDs) plus 4 kLux from the halogen lamps. The total irradiance is practically equal to bright sunlight conditions, but the continuous motion of the sources prevents the surface of the painting from being heated significantly (the maximum local heating produced by the scanning was measured at about 4 °C).

**Figure 1:**
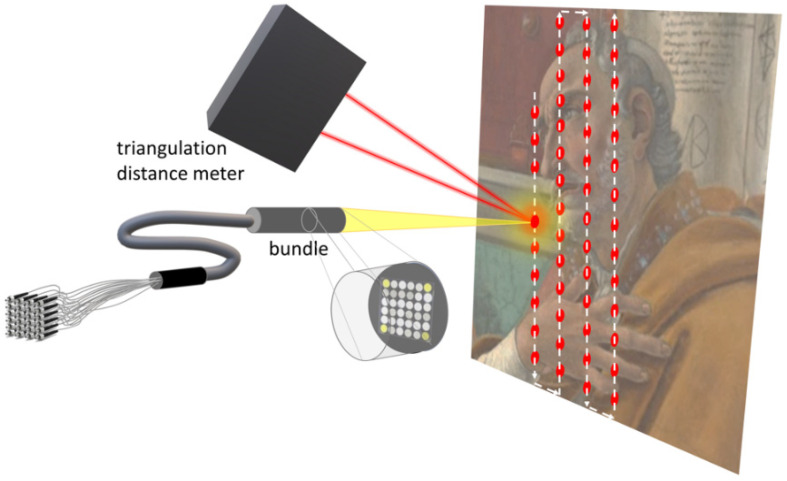
Experimental scheme for the synchronised VIS-NIR imaging spectroscopy and 3D sensing.

The image of the painting is acquired by X-Y scanning of the entire surface. For each point, a small square grid (6 × 6 pixels) of the image is acquired by imaging a small square portion (1.5 mm × 1.5 mm) of the painting surface on the free end of a 36-fibres square-shaped optical bundle that carries the light to 36 separate detectors (Figure 1). The optical fibre ends in the bundle are packed in a square arrangement and are evenly spaced at the same pitch of the pixels in the final image, 0.25 mm, which in turn is both the pitch of the scanning system and the pixel size of the resulting image (square pixels of 0.25 mm × 0.25 mm). The hardware image registration is then easy to be obtained, as it is sufficient to shift the 36 acquired image planes by whole-pixel steps (multiple to 0.25 mm) to align the spectra acquired in each pixel. In this process, a margin of 5 pixel is lost, as along the image borders there is insufficient coverage to obtain complete spectra. The output is a registered set of images, the so-called multispectral cube, that can be seen either as a stack of monochromatic images (one for each channel) or as a collection of discrete spectra (one for each pixel, i.e., 4000 spectra in the image of one square meter of surface). The whole process is handled by the instrument software, which produces images having resolution of 4 pix/mm (or 101.6 dpi) at a rate of about 3 h/m^2^.

This type of scanning almost entirely avoids the geometric distortion of the acquired image, resulting in an overall geometric distortion at the image border of less than 1/1000 (less than 1 mm per each metre of the image). The multi-spectral image can then be also used for measuring the size of details on the surface, for example to compare the underdrawings between different paintings in search for the use of transfer media for the drawings.

**Figure 2:**
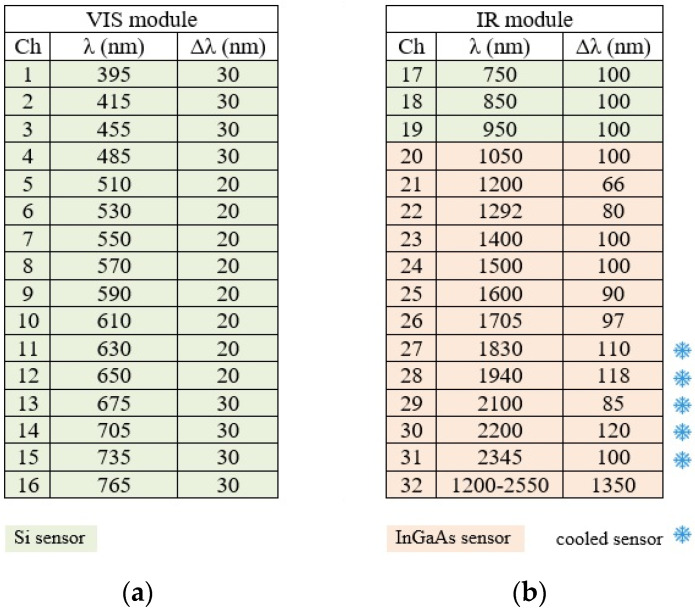
Central wavelengths and respective bandwidths of the 32 channels, 16 VIS (**a**) and 16 NIR (**b**) of the imaging spectroscopy device.

**Figure 3:**
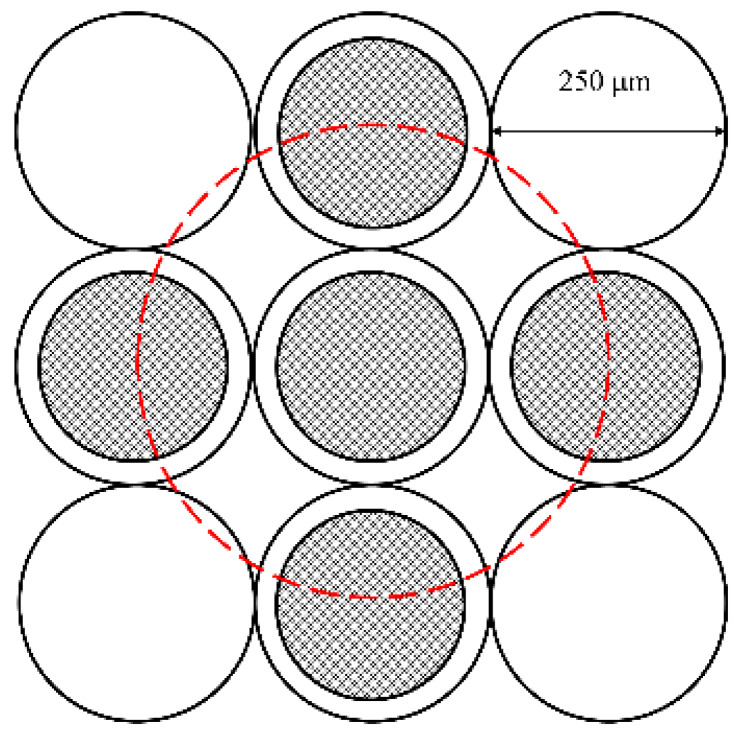
Detection scheme for spectra averaging over 5 pixels. The grey circles represent the fibre cores (200 μm); the dashed red circle is the averaging area.

Chromatic aberrations in the image, which could be expected considering the broad spectral region acquired (380–2500 nm), are actually avoided by using a fully catoptric optical system design, made by two off-axis parabolic mirrors arranged to give a unit (1:1) magnification. Transverse optical aberration of this system is not expected to be small, but in the specific set-up (paraxial usage), the field angle tended by the bundle half transverse size (0.75 mm at about 300 mm) is so small that all field aberrations are negligible, and the registration of the acquired images is aberration-free. The spectral bands are acquired with 32 separate photodetectors: 19 Si (380–1000 nm) and 12 InGaAs (1050–2500 nm) photodiodes, each equipped with a notch filter (multilayer interference filters) to select the acquisition spectral range. The filters have individual spectral resolutions ranging from about 20 to 30 nm in the visible (VIS) and from 60 to 120 nm in the near infrared (NIR), respectively. Central wavelengths and respective bandwidths are shown in Figure 2. The spectra extracted from the multispectral cube are reported as a mean value computed over a 0.5 mm circular area, i.e., over 5 pixels (Figure 3).

Another advantage of the combination of movable illumination, point-by-point scanning and detection with a catoptric optical system is that the resulting illumination is homogeneous through all the image, and between successively acquired images. This feature is very useful to get the image mosaicking of paintings wider than 1 m × 1 m.

Moreover, high-resolution single-point spectroscopy has been performed by directly coupling commercial spectrometers (Zeiss MCS 521 VIS-NIR-E and MCS-511 NIR 1.7, Carl Zeiss, Oberkochen, Germany) with the optical system of the VIS-NIR scanner. This gives the possibility of acquiring the detailed spectra in the very same conditions used for the multi-spectral scanning acquisition.

The autofocus (AF) system of the scanning device [27] is based on three elements: a high-speed triangulation distance meter, a motorized linear stage (the Z stage of the system, as previously described) and a custom-made control software, synchronized with the acquisition of the spectral reflectance image. The system runs by acquiring a set of profiles of the scanned surface, whose points are obtained by measuring the scanning probe-painting distance with the triangulation sensor (Figure 1), and generates a 3D map of the surface. The AF operates in an open-loop configuration, due to the speed of the scanning (approaching 500 mm/s) and, consequently, to the risk of having the optical head to move in an uncontrolled way. The open-loop system works by first acquiring the surface profile on the lines to be scanned (the vertical columns in the image), then calculating the profile to be fed to the AF stage and, finally, by generating the exact profile of motion for the Z stage, which is synchronised to the vertical scanning by using the signal of the vertical encoder on the Y stage. The Z stage moves with minimum steps of 50 microns and a maximum speed of 25 mm/s. By using this open-loop strategy, the Z stage moves in a totally controlled (and safe) way and adapts the optics-to-painting distance during the vertical scanning with a high accuracy. Being the depth of field of the acquisition optics about ±1 mm, the system is able to survey highly bended surfaces, that is often the case when dealing with ancient panel paintings, keeping the optical head at the exact working distance. Calculated motion profiles for the AF are previously fitted to the real surface profile so that the AF system is not sensitive to abrupt variations in the stand-off distance, e.g., presence of cracks, steps, frames, etc.

As a very useful by-product of the AF system, the 3D map of the painting surface is extracted from the raw distance data, as measured by the triangulation meter. The sensor provides a distance signal (analogue, ranging from 0 to 10 V) which is digitised by the scanner main ADC. The sampling rate, triggered by the Y stage encoder, is the same for all the sensors (the VIS, the NIR and the profile) so that the profile 3D map has the same raster resolution of the spectral images and is perfectly aligned to these. This integration makes it possible to extend the possible metrology of distances on the surface to a full 3D. The final image obtained has then the same properties of an orthophoto. Indeed, the autofocus system, synchronized with image set acquisition, generates a series of profiles, whose points are obtained by measuring the probe-painting distance. Such profiles were used to produce a 3D model of the surveyed surface (250-micron sampling step).

### 2.2. Examined Artwork

*Sant’Agostino nello studio* is a mural painting by Sandro Botticelli (2750 × 1750 mm) dating back to 1480. The fresco depicts Saint Augustine, the forerunner of the humanistic scholars, in his study. A great care was given to the description of the details, inspired by the realism of Flemish painters [28]. The artwork was originally located at the entrance wall to the choir in the church of *Ognissanti* (Florence, Italy) which was destroyed in the sixteenth century during the reconstruction of the church. The artwork was anyway already been detached *a massello* (detachment of a wall painting including several centimetres of the mural support) and transferred to the church nave by Giorgio Vasari. During the transfer, parts of the architectural frame and the inscription at the top of the Botticelli’s fresco were lost and possible alterations were made to the coat of arms of the Vespucci family, patron of the church. Following the flood in 1966, which affected the lower part of the fresco, the artwork was finally detached with the *stacco* technique from the original mural structure. The *stacco* technique consists in preserving only millimetres of the original pictorial plaster and attaching it to a new support, in this case, made of polyester resin and glass fibre. The artwork has been subject of numerous exhibitions all over the world, thanks to its transportability, and has recently been under restoration in the laboratories of the *Opificio delle Pietre Dure* in Florence [29]. On that occasion, the non-invasive analyses presented in this paper were carried out as well as other complementary analysis for robust material identification such as X-ray fluorescence (single-point and mapping), fibre optics reflectance spectroscopy, Fourier-transform infrared spectroscopy, etc.

## 3. Discussion and Results

The autofocus output, after being mapped in a mesh format, can be processed with image processing and/or 3D-handling software. The 3D map in a 2D image format can be handled so to create a shading effect that makes the surface look like a real one, except for its colour appearance, making morphology seemingly pop out from the screen. This relief effect (raking-light effect) is achieved through first derivative of the surface profile map.

**Figure 4:**
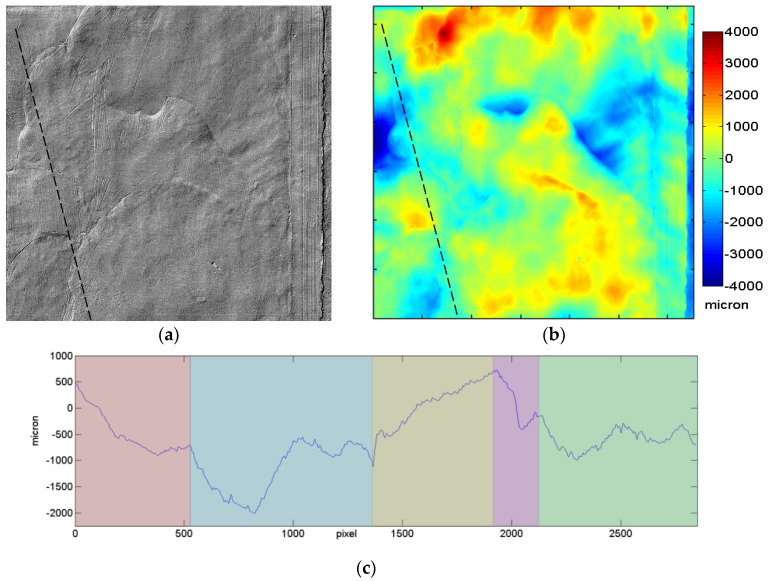
Detail of the Sant’Agostino nello studio fresco: simulated raking light image (**a**), conditioned surface colour map (**b**) and profile (**c**) along the dashed line in (**b**). The coloured background highlights the five regions corresponding to the *giornate*. Both (**a**) and (**b**) images are 3189 × 3269 pixels.

**Figure 5:**
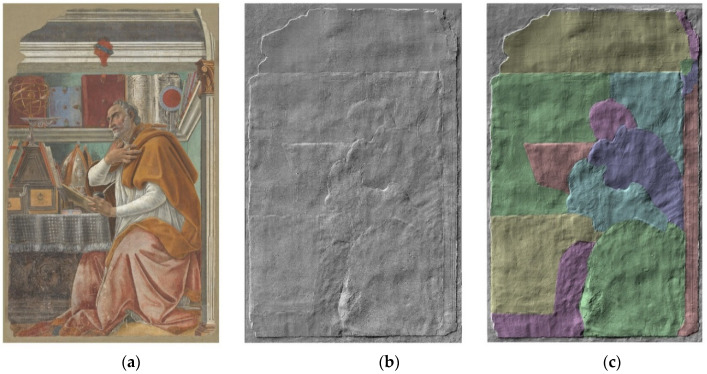
*Sant’Agostino nello studio* (2750 × 1750 mm^2^): RGB image (**a**) and 3D model displayed as a raking light image (**b**) with 14 evidenced *giornate* (**c**).

By a quantitative analysis of 3D data, it was possible to discern fourteen areas identifiable with the so-called *giornate*, corresponding to portions of the fresh plaster presumably worked in one day [30,31]. 3D raw maps were fitted with a best plane, which was subtracted to generate the so-called conditioned surface. A set of profiles was then acquired on such surface, along lines among the different areas and following different directions, to quantify the step heights. As an example, the profile along the black dashed line (Figure 4a,b), crossing five different *giornate*, is reported in Figure 4c. The coloured *giornate* image of the entire fresco was derived (Figure 5c) by merging the information from the simulated raking light image and the colour height map (where altitude is displayed as colour gradient), and the profile plot. Each working day of painting (*giornata*) has been coloured in a different shade.

Figure 5a shows the true-colour image produced by processing the visible region spectral dataset acquired with the multispectral scanner, collected in a 45°/0° illumination/detection geometry, by applying standard D65 illuminant and the CIE 1931 standard observer. Figure 5b displays a 3D model of the fresco, shown as a raking-light image, that is perfectly co-registered with the colour image. To provide a visual guidance, the coloured *giornate* in Figure 5c show, for example, that Botticelli has realized the saint’s head in one small stand-alone portion, a proof of his great care dedicated to the detail. Moreover, Figure 5c displays damage/abrasion in the zone of robe (green *giornata* lower right).

**Figure 6:**
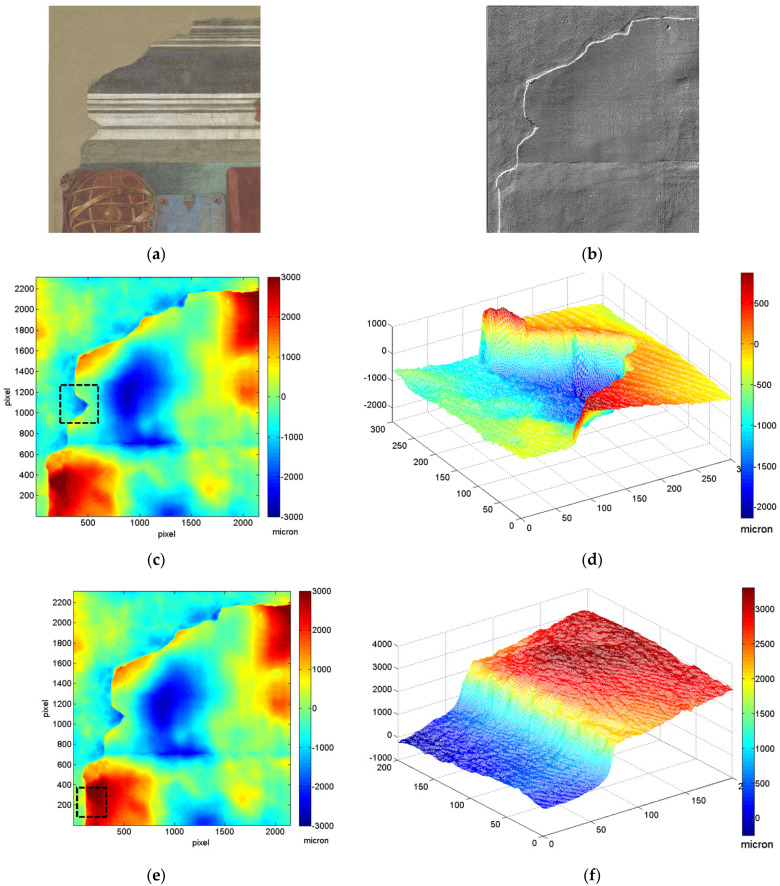
Upper left detail of the fresco: RGB and simulated raking light image (**a**) and (**b**); colour map displayed as a 2D image (**c**,**e**) and a 3D mesh (**d**,**f**).

The 3D model documents the conservation intervention performed in the 1970s. Following the flood in 1966, the fresco was detached from the original wall through the *stacco* process and placed on a new movable support. The latter is rendered in grey colour in the 3D model (Figure 5b). In Figure 6, a detail showing the step between the neutral plaster and the fresco is reported. 3D data enable to quantify the thickness of the original protruding material comprising both the pictorial layer(s) and the plaster to be about 1–3 mm, as evident from the 2D colour map images (Figure 6c,e) and 3D mesh plots (Figure 6d,f). Such conservation approach, in which the new neutral plaster is not at the level of the original fresco, is typical for that period.

**Figure 7:**
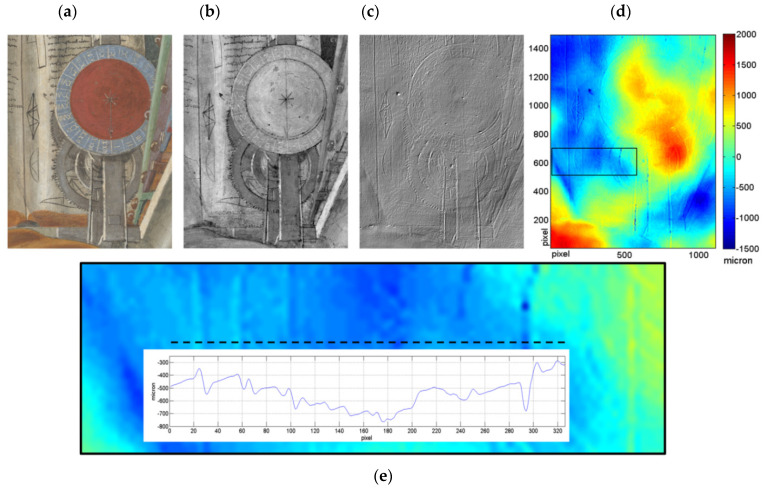
Detail of the clock: (**a**) RGB image; (**b**) reflectogram centred at 1700 nm, (**c**) simulated raking light image (**d**) colour map and (**e**) zoom in of the rectangle evidenced in (**d**) showing the in-depth profile plot acquired along the dashed line.

The VIS-NIR spectral data, examined as a set of 32 16-bit monochromatic images, were exploited to shed light on the technique used to draw the fresco. Among the common methods is the so-called *spolvero* that consists in tracing a natural-scale drawing on the paper then transferring it on the plaster by charcoal (carbon) powder diffused through tiny-punctured holes pierced in the paper along the drawing lines. As a result, rounded carbon-based signs remain on the freshly cast intonaco. Long-wavelength IR reflectograms (at wavelengths about 2000 nm) give good transparency of top layers (RGB detail of clock in Figure 7a) and provide evidence for *spolvero* (Figure 7b), due to the sufficient contrast between the carbon dots, absorbing the IR light, and the plaster substrate that reflects it.

Another way to transfer the drawing from paper to the plaster is to imprint the drawing on the fresh plaster by etching incisions along the drawing lines. These signs, usually printed by sharp metal instruments into the fresh plaster, leave a clear trace with raised edges [30,31], particularly visible in the simulated raking light image (Figure 7c). The colour map of the same area is shown in Figure 7d. The black rectangle indicates the position of an in-depth horizontal profile extracted (Figure 7e). Here a high-resolution 3D analysis was used for quantifying the thickness of the grooves of the engravings, resulting in a depth range from a few tens to one hundred micron, and width of nearly 10 microns.

Figure 7b reveals a *pentimento*, i.e., a change by Botticelli to his original drawing made during the realization stage. The reflectogram evidences a sketch of two clock hands in the preparatory phase: one pointing up (numeral XII) and the other down (XXIIII). Only the latter, indicating the sunset, was realized both with incision (Figure 7c) and in the successive pictorial process (Figure 7a). This could be either related to importance of the twilight in the fifteenth century indicating that all the residents need to enter the city because the city wall gates would close, or could be inspired by an iconographic interpretation [29].

The main pigments used in the fresco palette were identified through their spectra generated from the high spectral resolution Fibre Optics Reflectance Spectroscopy (FORS) and VIS-NIR dataset, and their distribution was mapped with spectral correlation maps (SCM). Basing on the measure of the spectral similarity between the spectrum of each pixel in the multispectral data and a specified reference spectrum (endmember), the SCM classification algorithm identifies different regions or materials in the multispectral data cube by computing the spectral angle distance between each pixel and the endmember spectra of the data cube, provided that the data is normalized and centred on the average of the two spectra. The spectral correlation mapper method is a modification of the Spectral Angle Mapper (SAM, one of the leading classification methods, which consists in obtaining the angles formed between the reference spectrum and the image spectrum treating them as vectors in a space with dimensionality equal to the number of bands), and is a derivative of Pearson’s correlation coefficient [32,33].

The latter is known as the best method of measuring the association between variables of interest because it is based on the method of covariance, giving information about the magnitude of the association, or correlation, as well as the direction of the relationship. Given a pair of random variables (X, Y), the formula for Pearson’s correlation coefficient, commonly represented by the Greek letter ρ, is

$$\rho_{X,Y}=\;\frac{cov\left(X,Y\right)}{\sigma_{X}\sigma_{Y}}=\frac{\sigma_{XY}}{\sigma_{X}\sigma_{Y}}$$

where *cov* is the covariance, $\sigma_{X}$ is the standard deviation of *X*, $\sigma_{Y}$ is the standard deviation of *Y*. Coefficient values can range from +1 to −1, where +1 indicates a perfect positive relationship, −1 indicates a perfect negative relationship, and a 0 indicates no relationship exists.

Pearson’s correlation coefficient, when applied to a sample, is commonly represented by $r_{xy}$ and may be referred to as the *sample Pearson correlation coefficient*. The formula for $r_{xy}$ is obtained by substituting estimates of the covariances and variances based on a sample into the formula above. Given paired data $\left\{\left(x_{1},y_{1}\right),\left(x_{2},y_{2}\right),\ldots ,\left(x_{n},y_{n}\right)\right\}$ consisting of *n* pairs, $r_{xy}$ is defined as:

$$r_{xy}=\frac{\sum_{i=1}^{n}\left(x_{i}-\bar{x}\right)\left(y_{i}-\bar{y}\right)}{\sqrt{\sum_{i=1}^{n}{\left(x_{i}-\bar{x}\right)}^{2}{\left(y_{i}-\bar{y}\right)}^{2}}}$$

where *n* is the sample size, $x_{i},y_{i}$ are the individual sample points indexed with *i*, and

$$\bar{x}=\frac{1}{n}\overset{n}{\underset{i=1}{\sum }}x_{i}$$

(sample mean), and analogously for $\bar{y}$.

**Figure 8:**
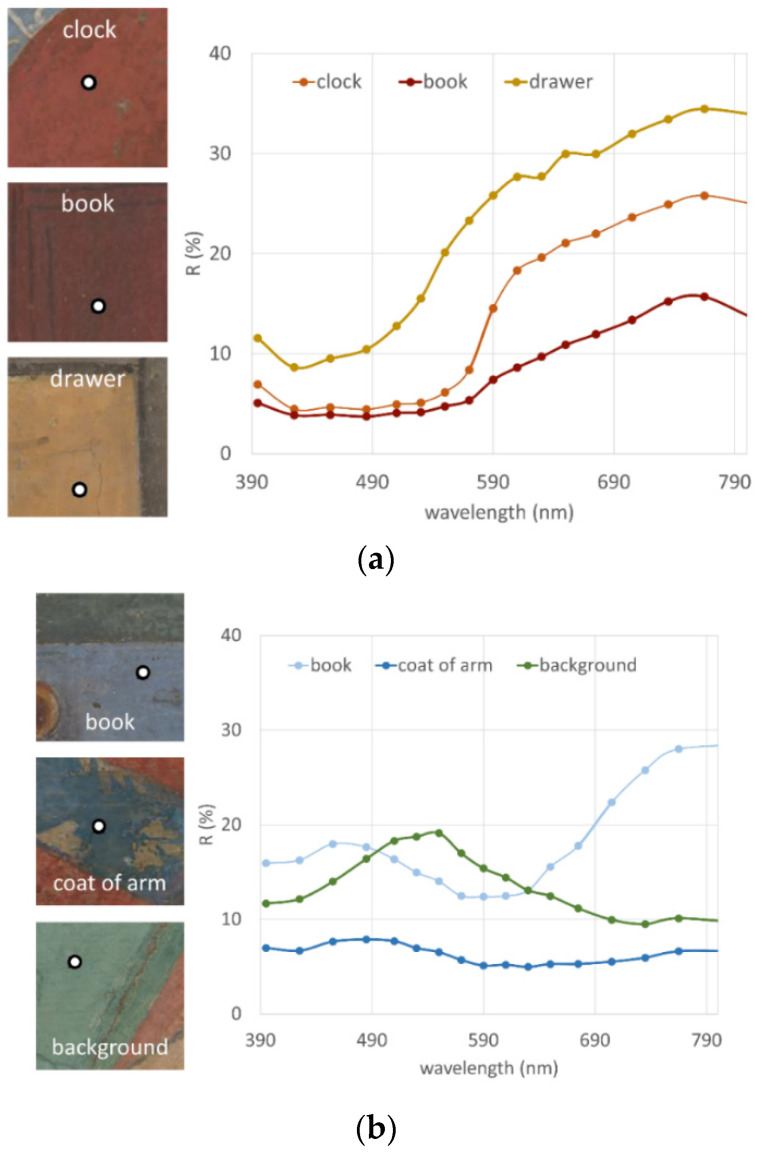
Reference endmember spectra for (**a**) the warm and (**b**) the cold colour shades. On the left, the white circle with black contour indicates the exact positioning of the sampled point.

For the red/yellow and blue/green hues, three representative reference spectra were chosen, as shown in Figure 8a,b. Those endmember spectra are the mean value computed over a 0.5 mm circular area, i.e., over 5 pixels (Figure 3). 

For each endmember spectrum shown in Figure 8, an SCM image was then generated. The so obtained SCM images were combined with the trichromatic modality. Specifically, the respective SCMs were attributed to the R, G, and B channel, for both Figure 8a (the clock, drawer and book spectrum) and Figure 8b (the coat of arms, book and background spectrum).

**Figure 9:**
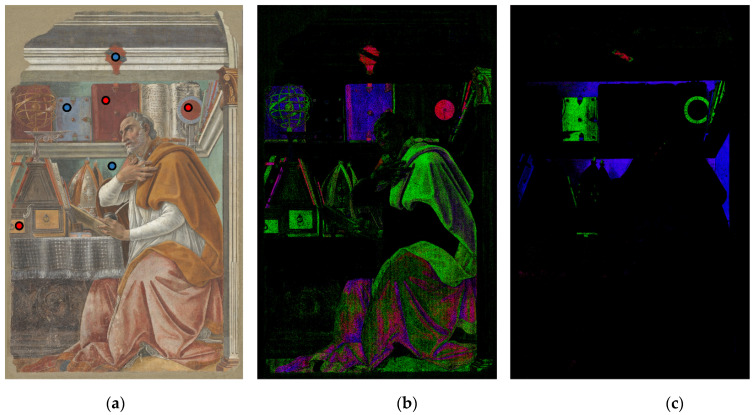
Fresco by Botticelli: RGB image (**a**), colour composite images from SCMs for warm in (**b**) and cold (**c**) hues. Sampled 0.5 mm areas for spectral endmembers are indicated in (**a**) with red and blue circles related respectively to (**b**) and (**c**). Figure 9b: red relates to SCM of “clock”, green to “drawer”, blue to the “book”, spectral endmembers in Figure 8a; Figure 9c: red relates to SCM of “coat of arms”, green to “book” and blue to “background” spectral endmembers in Figure 8b.

The visible image (Figure 9a) indicating the sampled points for the warm and cold hues endmember spectra, and the SCM resultant two false colour images are shown in Figure 9b,c. The chief advantage of this approach is that it generates trichromatic composite image concentrating all the information obtained with the SCM analysis. Concerning the Figure 9b, there is a strong correlation between the pigment used for the coat of arm and the clock (appearing in red), whereas the red pigment of the books is different (appearing mostly blue with violet binding). The saint robe is a mixture of pigments (magenta), instead the mantle, the drawers and the capital are nearly the same colour (appearing green), suggesting a single pigment type. The SCM image in Figure 9c shows a strong similarity between blue pigments of the small book and the clock.

Related to blue zones, two different pigments were discriminated through complementary analysis: prevalent ultramarine and smalt [29]. The SCM computed on VIS dataset shows the distribution of the ultramarine, coded in green colour. The map reveals its presence in the clock and the blue cover of the precious book. The ultramarine pigment, chemically the most complex of all the mineral pigments, is extracted from Lapis Lazuli stones imported in the past mostly from Asia. A broad absorption band at 600 nm (due to the charge transfer within the sulphur anions trapped in the aluminosilicate network) in VIS-NIR reflectance spectrum serves as its identifying marker, along with high reflectance values in red spectral region typical of natural ultramarine [34,35,36].

The second blue pigment distinguished through the VIS-NIR spectra is a cobalt based pigment. As the SCM evidences, its distribution is located within the coat of arms of the Vespucci family—the patron of *Ognissanti* church. The spectral pattern is compatible with cobalt-based pigments where two broad absorption bands—the first in 550–650 nm and the second in 1200–1550 nm range—origin from the electronic transitions among d orbitals of Co(II) ion in tetrahedral coordination with respect to oxygen atoms. One of these is the smalt (a potash silicate glass coloured with cobalt) [36,37].

Table 1 summarizes the discussed spectra and prevailing materials which are the main constituents of the fresco palette. As to the common practice [19], the material identification was supported by several complementary techniques, including FORS and X-ray fluorescence (single point and mapping) spectroscopy [29].

## 4. Conclusions

All the image properties discussed (image resolution and contrast, even illumination, spectral and metric accuracy) would have been impossible to obtain without a system to keep the exact working distance of the optical head to the scanned surface. There are many reasons for this: first the small field depth of the collecting optics (about ±1 mm) which requires that the surface-to-objective distance is kept within that depth during the whole scanning. Second, the uniformity of illumination and the spectral accuracy are greatly affected by small variations of the same focus distance. The autofocus (AF) system is then key to the scanner performances and to the syncing of the different sensing techniques. Moving the whole optical head to closely follow the surface profile while rapidly scanning requires the fast displacement of a considerable mass and, consequently, specially designed hardware and software systems.

The synergic multi-sensor approach in the investigation of a work of art by means of multispectral VIS-NIR analysis combined with topographic analyses provides evidence on the artist’s realization technique. The hardware-registered data enable straightforward data analysis bypassing the necessity for data fusion. Data from the 3D sensor allowed to extract information related to the *giornate*, *spolvero* and direct incisions. Spectral VIS-NIR data were used to map pigments of the masterpiece. Moreover, we have been able to discriminate the presence of a *pentimento* and to determine the composition and distribution of some later restoration intervention. The combined use of spectral imaging with 3D acquisition has been of great importance to gain insights on crucial features of the painting, also thanks to the portability and non-invasiveness of the coupled techniques.

The main advantage of the employed VIS-NIR instrument as compared to analogous devices is that data are aberration-free and hardware registered, where no correction and alignment post-processing is necessary. The twofold nature of the obtained data, in the spectral and the spatial domains, consents for the mapping of the materials constituting the artworks, such as the pigments and retouches, pentimenti, underdrawing, etc., maintaining a geo-referenced visual approach to the data analysis. The main benefit of the home-built scanner is its full controllability, versatility from both the analytical and the mechanical point of view. Synchronized VIS-NIR and 3D imaging supplies the experts with information that can be crucial about the most suitable restoration process and about the artwork’s realization and conservation history.

## Figures and Tables

**Table 1:** Hues and materials of the fresco as measured on the six points shown in Figure 8 and Figure 9.

Zone	Hue	SCM Colour Code	Materials
Clock	Reddish	Red (Figure 9b)	Prevailing cinnabar (α-HgS)
Drawer	Yellowish	Green (Figure 9b)	Yellow ochre (mixture of iron oxyhydroxide (FeOOH) and varying amounts of clay and sand)
Book	Brownish	Blue (Figure 9b)	Prevailing red ochre (mixture of iron oxide (α-Fe_2_O_3_) and varying amounts of clay and sand)
Coat of arms	Darker bluish	Red (Figure 9c)	Prevailing smalt (potassium glass containing cobalt oxide)
Small book	Bluish	Green (Figure 9c)	Ultramarine (approx. (Na,Ca)_8_(AlSiO_4_)_6_(SO_4_,S,Cl)_2_)
Background	Greenish	Blue (Figure 9c)	Green earth K[(Al,Fe),(Fe,Mg](AlSi_3_,Si_4_)O_10_(OH)_2_

## Data Availability

The datasets used in this study are available under request and following permission by the artwork owner. Please address requests for data reuse in writing to Raffaella Fontana (raffaella.fontana@ino.cnr.it).
